# Venous Cannulation Pain as a Marker of Postoperative Pain Vulnerability: A Pre-Specified Secondary Analysis of a Randomized Controlled Trial

**DOI:** 10.3390/jpm16010058

**Published:** 2026-01-22

**Authors:** Anna K. M. Persson, Krister Mogianos

**Affiliations:** 1 Department of Anesthesiology and Intensive Care Medicine, Halland Hospital Halmstad, 301 85 Halmstad, Sweden; krister.mogianos@med.lu.se; 2 Department of Clinical Sciences Malmo, Lund University, 205 02 Malmo, Sweden

**Keywords:** venous cannulation, postoperative pain, pain prediction, individualization, pain sensitivity

## Abstract

**Background**: Identification of patients at risk and prevention of acute postoperative pain (APOP) are central to individualized anesthesia and analgesia. Venous cannulation pain (VCP) has shown promise as a predictor of APOP. In the PeriOPerative Individualization Trial (POPIT), VCP was evaluated as a pain-sensitivity stratification method to guide anesthesia and reduce postoperative pain. This report presents a predefined secondary analysis with the primary aim to evaluate VCP as a method for postoperative pain prediction. As a secondary aim, we sought to explore factors that influence VCP. **Methods**: 271 patients were stratified into two cohorts, high-risk (VCP ≥ 2.0) and low-risk (VCP < 2.0), for APOP, based on their VCP. Within each group, patients were randomized to receive either: standard care or opioid-free anesthesia (low-risk cohort), and standard care or multimodal anesthesia with opioids (high-risk cohort). Differences in acute and persistent pain, quality of recovery, postoperative opioid consumption, and proportion of patients experiencing moderate to severe APOP depending on VCP levels were investigated. The predictive capacity of VCP was evaluated and adjusted for in terms of potential confounders. **Results**: High-risk patients, grading VCP ≥ 2.0 (VAS units) experienced more APOP on the day of surgery (difference 0.9 NRS-units, 95% CI 0.2–1.6, *p* = 0.009) and after 24 h during movement (difference 0.6 NRS-units, 95% CI 0.0–1.3, *p* = 0.048). Patients grading VCP < 2.0 had better quality of recovery after 24 hr (difference 7, 95% 1–13, *p* = 0.002) and lower postoperative opioid consumption (difference 7.5 mg, 95% 5.7–9.3, *p* < 0.001). The OR for VCP ≥ 2.0 to predict APOP in PACU was 1.76 (95% CI 1.02–3.04, *p* = 0.043), but in a multivariate model, adjusted for age, VCP ≥ 2, gender, pain catastrophizing, preoperative pain, and pain on the day of surgery, female gender was the only independent predictor of APOP (OR 2.65 (95% CI 1.33–5.29), *p* = 0.006). **Conclusions**: Pain during venous cannulation as a predictor of acute pain after surgery was significant in univariate regression, but the results were lost when adjusting for confounders like gender and current pain. However, VCP continues to show potential in associated postoperative recovery outcomes such as opioid consumption. The level of pain associated with venous cannulation is influenced by gender, preoperative pain, and current pain on the day of surgery. Pain sensitivity stratification needs refinement before implementation in clinical practice.

## 1. Introduction

Prevention and treatment of acute postoperative pain (APOP) is, despite many years of scientific interest, an ongoing challenge. Traditionally, procedure-specific treatment protocols have been used, but the focus is shifting towards individualized therapeutic strategies to manage APOP, postoperative recovery, and persistent postoperative pain (PPOP) [1,2]. Risk factors for APOP include female gender, younger age, pre-operative pain, psychosocial factors (e.g., anxiety and depression), certain types of surgery, specifically surgery with nerve damage, and large skin incision [3,4,5,6]. Various preoperative screening methods have been proposed to help predict the risk of APOP. These include psychometric evaluations, quantitative sensory testing (QST), serologic biomarkers, genetic profiling, and real-time perioperative monitoring. Integrative models combining multiple risk factors are also being explored. However, none of these methods have yet demonstrated sufficient predictive accuracy and feasibility for routine clinical use [7,8,9].

Pragmatic approaches have been developed to assess pain sensitivity during routine preoperative procedures. These include tourniquet-induced pain and pain from subcutaneous local anesthetic injection before spinal anesthesia [10,11]. Similarly, pain associated with peripheral venous cannulation, “venous cannulation pain” (VCP), has been linked to earlier requests for epidural analgesia in obstetric patients [12] and has also been shown to predict acute pain in the perioperative setting. In a study on elective laparoscopic cholecystectomies, pain-sensitive patients grading their VCP above 2.0 VAS units had 3.4 times higher odds of moderate to severe APOP [13]. Similarly, in a mixed group of patients undergoing different surgical procedures, the OR for pain-sensitive patients (VCP ≥ 2.0) was 1.7 [14]. These results were further confirmed in an independent cohort undergoing laparoscopic nephrectomy, showing that VCP predicted moderate to severe APOP with an OR of 3.5 and a strong correlation with APOP (r_s_ = 0.64) [15]. VCP is a quick, intuitive method and merits further investigation as a tool for individualized perioperative pain management.

This study was a pre-specified secondary analysis of the PeriOPerative Individualization Trial (POPIT), which aimed to improve pain outcomes by individualizing anesthesia for patients in a randomized, controlled study based on a stratified risk for postoperative pain. Here we present data in terms of VCP as a stratification method and explore its use in individualized anesthesia. The primary aim of this pre-specified secondary analysis was to evaluate VCP as a method for postoperative pain prediction when accounting for potential confounders. Our secondary aim was to evaluate factors that may influence VCP. We present results on persistent pain for the first time and associations with other preoperative risk factors like gender, age, psychometric variables, and preoperative pain.

## 2. Methods

### 2.1. Study Design and Ethics

The POPIT is a randomized, controlled, patient- and assessor-blinded, single-center, parallel-group clinical trial. The protocol was approved by the Swedish Ethical Review Authority (approval number 2020-06178) and complies to the ethical principles of the declaration of Helsinki. This manuscript was written in accordance with the Consolidated Standards of Reporting Trials (CONSORT) and was also pre-published in ClinicalTrials.gov (ID NCT04751812). In favor of total transparency, the study protocol and methods have been pre-published. As a pre-specified secondary outcome, the ability of VCP to predict APOP and PPOP and its relationship to other perioperative risk factors were the focus of this study.

### 2.2. Patient Recruitment

The recruitment and data gathering process was ongoing between March 2022 and February 2024.

Inclusion criteria were elective laparoscopic surgery, age > 18 years, American Society of Anesthesiologist (ASA) class < III, and the ability to understand and accommodate information in Swedish. Exclusion criteria were refusal to participate or withdrawal of consent, pregnancy, allergy towards NSAID, and conversion to open abdominal surgery.

### 2.3. Preoperative Evaluation

Self-reported information regarding age, gender, psychometric evaluation, and preoperative pain were collected preoperatively. For psychometric evaluation, we used the pain catastrophizing scale (PCS), a questionnaire with 13 questions measuring the negative orientation around the thought of pain [16]. Participants rate statements on a scale of 0 (not at all) to 4 (all the time), with a maximum score of 52. A few recent studies have correlated the PCS to the intensity of APOP [17,18] and PPOP [19,20]. Patients were also asked about depression or ongoing treatment for depression (yes/no).

Patients were asked to state the presence of preoperative pain lasting more than 3 months (yes/no), preoperative pain during the last 2 weeks (yes/no), as well as the usage of analgesics (yes/no). Upon arrival at the preoperative preparation unit, patients were asked to grade their current pain on the day of surgery (NRS units).

### 2.4. Venous Cannulation Pain

All patients received an intravenous cannula, either at the dorsal aspect of the hand or antecubital fossa on the upper extremity, by a nurse with at least 3 years of working experience. After venous cannulation, patients were asked to grade their pain associated with the successful procedure on a handheld visual analog scale (VAS) where far left was described as no pain and denoted 0.0, and far right was described as the worst pain imaginable corresponding to 10.0. Patients grading their VCP < 2.0 were stratified as “low-risk”, while patients grading their pain as VCP ≥ 2.0 were considered “high-risk”. Thus, low-risk and high-risk cohorts were created from the initial sample. The cut-off (VCP < 2.0/VCP ≥ 2.0) was derived from previous independent studies concluding this to have promising predictive performance [13,14,15].

### 2.5. Anesthesia Protocol

Patients’ risk stratified as “high-risk” (VCP ≥ 2.0) were randomized to receive anesthesia either as standard of care (SOC) or multimodal anesthesia (MAO). Patients’ risk-stratified as “low-risk” (VCP < 2.0) were randomized to either SOC or opioid-free anesthesia (OFA). Details in terms of anesthesia protocols can be found in the pre-published protocol article. The randomization process was conducted in a 1:1 fashion for each cohort. All were given premedication with paracetamol, etoricoxib, and ondansetron. All but the OFA group were also given preemptive oxycodone. Maintenance of anesthesia were facilitated with a combination of sevoflurane/remifentanil or propofol/remifentanil (SOC and MAO) or sevoflurane/esketamine/dexmedetomidine (OFA). Before terminating general anesthesia, postoperative analgesia was given at a dose of 0.1 mg/kg oxycodone iv for SOC and MAO groups. Perioperative anti-nociception with esketamine and clonidine iv was given in the MAO group. The OFA group was given dexmedetomidine, esketamine and lidocaine as intraoperative anti-nociception.

### 2.6. Outcomes

Outcomes in this study were pre-specified as secondary outcomes in the POPIT. The main outcomes were the differences in worst APOP in the PACU and after 24 h at rest and during movement (APOP_POD0_, APOP_POD1rest,_ APOP_POD1movement_), as well as the differences in PPOP after 3 and 6 months at rest and during movement (PPOP_POD90rest_, PPOP_POD90movement_, PPOP_POD180rest_, PPOP_POD180movement_), between “high-risk” and “low-risk” patients in both the full cohort and the SOC-only cohort. Furthermore, the proportions of patients experiencing moderate to severe pain (NRS ≥ 4) in the PACU and after 24 h, the differences in postoperative opioid consumption, and the quality of recovery (QoR_POD1_) between “high-risk” and “low-risk” patients in both the full cohort and the SOC-only cohort were compared. Postoperative opioid consumption was defined as the sum of intraoperatively given oxycodone, before terminating general anesthesia, and postoperative rescue opioids until discharge from the post anesthesia care unit (PACU). This was measured using intravenous morphine milligram equivalents (MME), calculated by multiplying the intravenous dose of oxycodone by 1.5. Since only short-acting opioids (remifentanil) were used intraoperatively, this was not considered as postoperative opioid consumption.

### 2.7. Statistical Analysis

The full cohort from the POPIT was analyzed in this study. Since the main purpose was to assess VCP as a preoperative method to predict risk of APOP, patients in each cohort were clustered to obtain higher power and more robust data. As presented in the results of the POPIT, there was no difference in pain outcome between treatment arms in the two cohorts justifying the use of all patients in this study [21,22]. However, we have presented an evaluation of the differences in the two standard of care arms.

As POPIT was a pragmatic interventional trial meant to be able to show a clinically significant difference in APOP, a sample effect of 1.5 NRS units was chosen as this is in line with what is clinically meaningful for patients [23,24]. Previous studies in our institution have shown a SD of 2.8 and 2.7 in the high-risk cohort and low-risk cohort, respectively [13]. Collectively, 280 patients were needed to answer the primary research question in POPIT and account for a 20% dropout rate.

For all outcomes, the goal was to detect superiority between cohorts. Thus, in outcomes based on continuous data, hypothesis testing was conducted using the two-sided Independent T-test. For dichotomized pain outcomes, cohort comparison was performed using four-by-four contingency tables and the Chi-Square test.

Lastly, the predictive capability of VCP was analyzed with a stepwise multivariate logistic regression model with moderate to severe APOP (NRS ≥ 4) as a dependent variable. Candidate predictors were selected based on clinical relevance (preoperative pain, age, BMI, gender) and prior ambiguous evidence from the literature (PCS). In addition, variables showing a potential association with the outcome (*p* < 0.1 in univariate analysis) were retained for multivariate modeling. In the final step, a significance level of *p* < 0.05 was selected to mark the variable as an independent predictor. With a similar modus, we also conducted a multivariate logistic regression, with VCP ≥ 2.0 as a dependent variable, to investigate which variables influenced its value. Since the focus in this study was to evaluate VCP, how it influences APOP, and in turn what influences this variable, the same candidate predictors were used as in the initial model for APOP. To address concerns about model overfitting, discrimination was assessed by the area under the ROC curve (AUC). The calibration was assessed by calibration-in-the-large (intercept), calibration slope (e.g., regressing the outcome on the model’s linear predictor), and the Hosmer–Lemeshow test. The coefficient stability was examined with BCa bootstrapping (1.000 resamples).

All statistical analyses were performed using IBM SPSS Statistics (version 29), with a significance level set at *p* < 0.05.

## 3. Results

**Figure 1:**
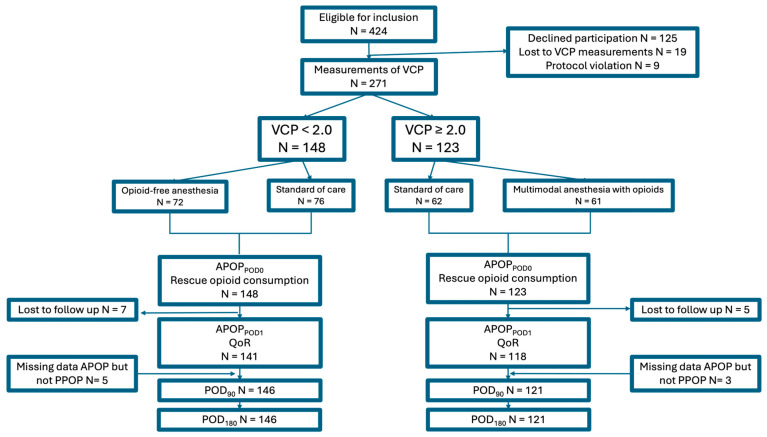
Inclusion flow-chart. Abbreviations: APOP; acute postoperative pain, POD; postoperative day, QoR; quality of recovery, VCP; venous cannulation pain, PPOP; Persistent Postoperative Pain.

In this pre-specified secondary analysis, a total of 271 patients were included. All of them were assessed for APOP and postoperative opioid consumption up to discharge from the PACU. On POD1, 12 patients were lost to follow-up, leaving 259 patients available for analysis of APOP_POD1rest_, APOP_POD1movement_, and QoR_POD1_. Of the previous 12 patients who were initially lost to follow-up on POD1, 8 were later able to provide data for PPOP at 90 and 180 days. (Figure 1). Background characteristics of the study population are summarized in Table 1 (Table 1).

### 3.1. Postoperative Pain

#### 3.1.1. Acute Postoperative Pain

**Figure 2:**
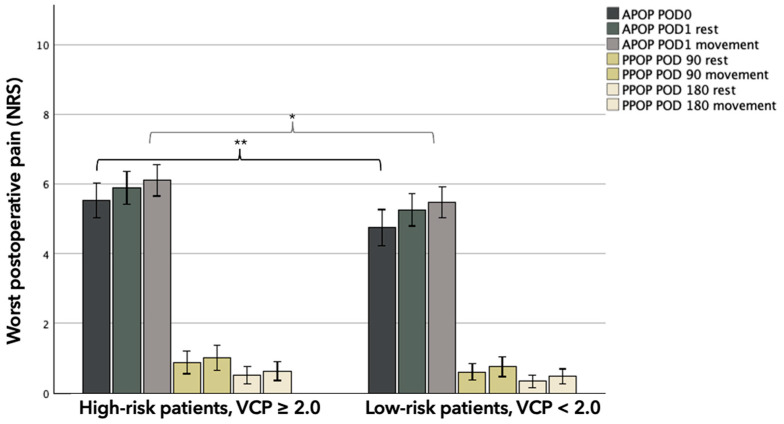
Bar chart depicting worst postoperative pain levels (NRS) grouped after levels of pain associated with venous cannulation. Whiskers represent 95% CI. Abbreviations: APOP; Acute Postoperative Pain, PPOP; Persistent Postoperative Pain, POD; postoperative day, NRS; numeric rating scale, VCP; venous cannulation pain. * Significance at 0.05 level. ** Significance at 0.01 level.

High-risk patients (VCP ≥ 2.0) reported higher levels of APOP (in the PACU and at POD1) compared to low-risk patients (VCP < 2.0), in both cohorts. Group-based difference in worst APOP_PACU_ were 0.9 (95% CI 0.2–0.6, *p* = 0.009) and 1.1 (95% CI 0.0–2.0, *p* = 0.047) NRS-units for the full cohort and SOC-only cohort, respectively. Worst APOP_POD1_ was consistently higher in the high-risk group for the full cohort, but a statistically significant difference was only found during movement (group-based difference 0.6, 95% CI 0.0–1.2, *p* = 0.048) (Figure 2). This pattern was consistent between SOC-only groups and statistically significant both at rest (group-based difference 1.2, 95% CI 0.2–2.1, *p* = 0.016) and during movement (group-based difference 0.9, 95% CI 0.1–1.8, *p* = 0.039) (Table 2).

#### 3.1.2. Persistent Postoperative Pain

PPOP at POD 90 and POD 180 did not differ significantly between the groups. The pain scores at rest and during movement were numerically higher in the high-risk group, but none of these comparisons reached statistical significance in either the full cohort or the SOC-only cohort (Table 2) (Figure 2).

#### 3.1.3. Risk Factors for Acute Postoperative Pain

A stepwise multivariate logistic regression for the full cohort was performed to identify independent predictors for moderate to severe APOP_PACU_ (NRS ≥ 4). In the univariate model, six variables were significantly associated with the outcome (Table 3). Subsequently, in the multivariate model, female gender was the only independent risk factor for moderate to severe APOP_PACU_ (OR = 2.65, 95% CI 1.33–5.29, *p* = 0.006). This association was consistent for moderate to severe APOP_POD1_, (female gender: OR = 2.20, 95% CI 1.05–4.56, *p* = 0.036). Additionally, at this timepoint, age reduced the odds for NRS ≥ 4 by 30% for each 10-year increase in age (*p* = 0.002), and PCS acted as independent risk factors for moderate to severe APOP_POD1_ (OR = 1.04, 95% CI 1.00–1.07, *p* = 0.045).

#### 3.1.4. Multivariate Model Performance

Among 271 patients, 193 (71%) had NRS ≥ 4. With six predictors, the events per variable was ~32. Apparent discrimination was AUC = 0.691 (95% CI 0.628–0.754, *p* < 0.001) and 0.738 (95% CI 0.679–0.797, *p* < 0.001) for moderate to severe APOP_PACU_ and APOP_POD1_. Calibration was acceptable for both time points: intercept ≈ 0.00 (95% CI −0.44–0.44) and calibration slope ≈ 1.00 (95% CI 0.60–1.40); Hosmer–Lemeshow *p* = 0.213 (APOP_PACU_), *p* = 0.459 (APOP_POD1_). In 1000 sample BCa bootstrapping, female gender remained a robust adjusted predictor, whereas age, pain- and PCS-related predictors were not robust after adjustment, which was consistent with collinearity among related constructs.

### 3.2. Venous Cannulation Pain

A multivariate logistic regression analysis was performed looking at factors potentially influencing the VCP level (Table 4). The model demonstrated acceptable calibration, as assessed by the Hosmer–Lemeshow goodness-of-fit test (*p* = 0.801). In the final model, preoperative current pain on the day of surgery (OR = 1.23, 95% CI 1.05–1.43, *p* = 0.008) and female gender (OR = 2.25, 95% CI 1.11–4.55, *p* = 0.024) were independent predictors for VCP ≥ 2.0, as was the pre-specified cut-off. Other variables, including age, PCS, and preoperative pain, were not significantly associated with VCP ≥ 2.0 in the multivariate model.

## 4. Discussion

In this study, VCP was associated with higher APOP and greater opioid consumption in unadjusted group comparisons. Patients with VCP ≥ 2.0 reported significantly more pain in the PACU and at POD1, and had better recovery scores, suggesting that nociceptive sensitivity, with VCP as a surrogate, may reflect a broader vulnerability to postoperative pain. However, when controlling for other variables in a multivariate regression, VCP did not remain an independent predictor for APOP, indicating that while preoperative VCP may be associated with worse pain outcomes, its predictive value is likely confounded by other factors, mainly female gender. However, VCP may reflect a preoperative pain-sensitive phenotype and provides a practical way to add nuance to how we phenotype patients who otherwise arrive in the operating theater as an undifferentiated group. From a clinical perspective, this means VCP should not be viewed as a standalone predictor but as part of a broader risk profile. While the effect size for VCP was modest after adjustment, its ability to capture a dimension of vulnerability remains relevant for early identification and individualized perioperative strategies. Furthermore, VCP’s ability to reflect individual pain sensitivity aligns with evidence supporting other preoperative pain prediction methods [7]. This suggests that VCP could complement existing strategies and help tailor perioperative pain management more effectively.

In regards to gender, since the cohort in this study consisted mainly of female patients (full cohort; 79%, SOC cohort; 82%) due to high proportion of gynecological procedures, the skewed sample does not exclude the possibility that gender in fact does not influence VCP. In this study, VCP ≥ 2.0 was more common among women (50% vs. 28%), and women had higher APOP_PACU_ incidence (77% vs. 52%). However, the small sample size in the male subgroups (n = 58, n = 16 with VCP ≥ 2.0) precludes us from drawing any certain conclusions about the interaction between gender and VCP. Other studies on VCP as a prediction tool did not find any gender-based influence on its outcome [13,14,15]. In the overall perioperative literature, female gender is widely accepted as an independent risk factor for postoperative pain [25,26,27]. For pain in general, 45% of women and 31% of men reported experiencing pain during their lifetime [28]. Before inferring casual inference, other factors such as biological (hormonal fluctuations), psychosocial factors (pain catastrophizing, cultural norms around pain expression), and measurement bias (differences in reporting behavior) must be further elucidated [29,30].

The differences in APOP depending on VCP levels presented in this study are smaller than in previous studies. Several factors may explain this. Earlier studies by Persson [13] and Peng [15] excluded patients with preoperative pain, whereas our cohort included many patients with significant pain before surgery. Since preoperative pain is one of the strongest predictors of postoperative pain, this likely influenced our results. Consequently, our cohort was heavily skewed toward high-risk patients for APOP.

The difference in APOP was smaller than what we have regarded as clinically meaningful, but we argue that the test’s easy accessibility, such as not demanding equipment or procedures not otherwise used, still adds value in the assessment of pain sensitivity.

Despite the cohort being high-risk, 148 patients were classified as low-risk and 123 patients as high-risk based on VCP. This imbalance raises questions about the sensitivity and specificity of VCP in this context. Specifically, in a high-risk cohort, sensitivity may be reduced if VCP misclassifies high-risk patients as low-risk, while specificity may be distorted due to the rarity of true low-risk cases. Future studies may need to recalibrate VCP thresholds for imbalanced cohorts or apply propensity score adjustments to balance covariates.

Unlike regression modeling (models the outcome), which focuses on estimating the effect of predictors on the outcome, propensity score methods (models the exposure) ensure that patients classified as high- or low-risk for APOP are comparable in terms of baseline characteristics. This was not pre-specified in our statistical plan. However, when performing a post hoc propensity score adjustment for the same variables used in the multivariate logistic regression model, VCP still did not act as an independent predictor. The propensity score was highly significant (*p* < 0.001), indicating that baseline covariates explain most of the variation in APOP risk.

Another difference is that, in this study, the location for cannulation varied. In previous studies with higher predictive values, the anatomical location for cannulation was limited to the back of the hand, which is known to be more painful. In another cohort of 505 patients, the VCP intensity was scored significantly lower in the antecubital fossa than on the back of the hand (1.0 vs. 2.0 VAS units, *p* < 0.001), and when combining sites of cannulation found an OR of 1.7 (95% CI 1.2 to 2.6, *p* = 0.005) for VCP ≥ 2.0 to render moderate to severe APOP [14]. Studies that measured VCP using cannulation at the back of the hand have reported the strongest associations, with odds ratios of 3.4 and 3.5, respectively [13,15]. One study failed to even show an association with VCP using only the back of hand (ß 0.88, 95% CI −0.18–1.94, *p* = 0.10) in a cohort of 102 patients undergoing knee arthroplasty in spinal anesthesia [31]. Both latter studies included patients with preoperative pain, whereas the study that failed to show an association only included patients receiving spinal anesthesia, which has previously been shown to influence results [14].

There are some limitations to this study. We included patients of both genders, but it turns out the cohorts were overrepresented by females. This is most likely related to the fact that we perform gynecological surgeries at our hospital but not urologic. However, there was no gender difference in other abdominal surgery types. The mean age was 55 years, which in this context is quite young. And no less than 47% answered yes to the question of having had preoperative pain lasting more than 3 months before surgery. At first glance, this might look like a typical cohort for major surgery, but in reality, it is relatively young, predominantly female, and characterized by high levels of preoperative pain. The small male subgroup (n = 58, only 16 with VCP ≥ 2.0) limits power to detect gender-based interaction effects. Given these findings, VCP’s unique predictive value appears limited once gender is accounted for. Modeling VCP as a continuous variable and evaluating incremental discrimination and calibration may clarify whether VCP adds meaningful predictive performance beyond established risk factors. Larger, gender-balanced studies are needed to confirm these observations.

Another limitation worth noting is the generally high levels of APOP observed in this study. In the PACU, 72% of patients experienced moderate to severe APOP, with mean pain scores of 5.0 (±3.0) NRS units. On POD 1 at rest, 69% reported similar pain levels, averaging 5.4 (±2.7). Persistent postoperative pain (PPOP) was present in 27% of patients at 3 months (NRS ≥ 1.0) and 19% at 6 months. Even though it is minimally invasive, laparoscopy is known to be associated with high APOP [6,32]. The skewness of gender and the high presence of preoperative pain may explain this, as the cohort can be considered as high-risk for APOP. We were also surprised by the overall low doses of opioids administered in the PACU. This will surely be something for us to improve in the future.

Although 271 participants were included in the multivariate analysis, the sample size may still be insufficient to fully support complex modeling. This could limit robustness and generalizability because confidence intervals for several predictors were wide, and some associations that were significant in the univariate analysis (e.g., all but gender) lost significance after adjustment. Such instability suggests reduced power to detect smaller effects and a higher risk that estimates may vary in other populations.

Finally, the difference in anesthesia and analgesia strategy has the potential to influence APOP due to different pharmacodynamic properties. It lies within every anesthesiologist’s belief that we can modulate outcomes with our pharmacological interventions. However, in the main study, which aimed to pharmacologically intervene upon APOP in different risk cohorts, there was no statistical difference between the groups [21,22]. Thus, we believe that the difference in pharmacological interventions in this sample did not contribute to significant bias in this study.

One of the main strengths of this study is its original randomized design, which provides strong internal validity by minimizing the risk of bias. Although this pre-specified analysis was secondary, the controlled nature of the trial reduces the likelihood of confounding factors, thereby strengthening the causal interpretation of the findings. In addition, the POPIT was designed as a pragmatic trial, enhancing its relevance and applicability to real-world clinical practice. Finally, the study protocol was published in advance and all outcomes in this study were pre-specified, reducing the risk of type I errors and increasing transparency.

## 5. Conclusions

In conclusion, VCP levels were able to predict risk for moderate to severe APOP with an OR of 1.8 in a univariate regression model, but, in this cohort, VCP’s predictive performance did not withstand adjustment for known clinical confounders, mainly driven by gender. Moreover, VCP was not effective in predicting PPOP. Current pain on the day of surgery and female gender influenced the pain associated with venous cannulation.

Future studies should aim to replicate these findings with a more gender-balanced sample. Furthermore, such studies should also explore whether different cut-off values for VCP and combining VCP with other risk factors might improve predictive accuracy in order to acquire a more robust model.

## Figures and Tables

**Table 1:** Preoperative data and group-based comparison between high-risk and low-risk groups in both the full cohort and standard of care only cohort, respectively.

	Full Cohort			SOC-Groups		
	High-Risk (VCP ≥ 2.0), *n* = 123	Low-Risk (VCP < 2.0), *n* = 148	*p*-Value	High-Risk (VCP ≥ 2.0), *n* = 62	Low-Risk (VCP < 2.0), *n* = 76	*p*-Value
Age, (SD)	53 (±16)	56 (±16)	0.099	53 (±16)	57 (±15)	0.146
Female gender, n (%)	105 (86.1)	107 (72.3)	0.006 **	55 (75.0)	57 (88.7)	0.040 *
Body mass index, kg/m^2^ (SD)	26.8 (±5.2)	26.5 (±4.6)	0.671	26.9 (±5.0)	28.6 (±24.2)	0.593
ASA, n (%)			0.800			0.639
I	50 (42.6)	59 (40.1)		24 (38.7)	32 (42.7)	
II	70 (57.4)	88 (59.9)		38 (61.3)	43 (57.3)	
Preoperative pain, n (%)	67 (54.5)	65 (43.9)	0.072	35 (56.5)	30 (39.5)	0.047 *
Current pain on day of surgery, NRS (SD)	1.8 (±2.4)	0.9 (±1.4)	<0.001 **	0.8 (±1.2)	1.9 (±2.5)	<0.001 **
Preoperative depression, n (%)	17 (13.8)	15 (10.1)	0.336	8 (12.9)	6 (7.9)	0.332
PCS, (SD)	15 (±10)	13 (±9)	0.046 *	15 (±10)	13 (±9)	0.321
VCP, VAS (SD)	3.7 (±1.7)	0.8 (±0.6)	<0.001 **	3.8 (±1.9)	0.7 (±0.6)	<0.001 **
Lap. rectum resection	7 (5.7)	16 (10.8)		6 (9.7)	9 (11.8)	
Lap. hemicolectomy	8 (6.6)	12 (8.1)		3 (4.8)	10 (13.2)	
Lap. colectomy	0 (0.0)	2 (1.4)		0 (0.0)	1 (1.3)	
Lap. sigmoideum resection	14 (11.5)	4 (2.8)		6 (9.7)	2 (2.6)	
Lap. cholecystectomy	28 (23.0)	42 (34.4)		16 (25.8)	19 (25.0)	
Lap. hernia repair	3 (2.5)	13 (8.8)		1 (1.6)	5 (6.6)	
Lap. adrenal resection	1 (0.8)	1 (0.7)		1 (1.6)	0 (0.0)	
Lap. ovarian cyst enucleation	9 (7.4)	5 (3.4)		4 (6.5)	2 (2.6)	
Lap. uni/bilateral salphingoophorectomy	21 (17.2)	24 (16.2)		11 (17.7)	13 (17.1)	
Lap. hysterectomy	31 (25.4)	29 (19.6)		14 (22.6)	15 (19.7)	

Abbreviations: SD; Standard deviation, ASA; American society of anesthesiologists’ physical status classification system, NRS; Numeric rating scale, PCS; Pain catastrophizing scale, VCP; Venous cannulation pain, VAS; Visual analog scale. Statistically significant differences are marked by an asterisk (*) in conjunction with the absolute value. * Significance at 0.05 level. ** Significance at 0.01 level.

**Table 2:** Postoperative outcomes and group-based comparison between high-risk and low-risk groups as group-based mean values with standard deviation.

	All Patients				Standard of Care Groups Only			
	VCP < 2.0	VCP ≥ 2.0	*p*-Value	95% CI	VCP < 2.0	VCP ≥ 2.0	*p*-Value	95% CI
APOP_POD 0_ NRS (± SD)	4.7 (3.1)	5.6 (2.7)	0.009 **	0.2–1.6	4.5 (3.1)	5.6 (2.8)	0.047 *	0.0–2.0
APOP_POD 1rest_ NRS (± SD)	5.3 (2.8)	5.9 (2.5)	0.063	0.0–1.2	4.9 (2.8)	6.1 (2.6)	0.016 *	0.2–2.1
APOP_POD 1movement_ NRS (± SD)	5.5 (2.7)	6.1 (2.4)	0.048 *	0.0–1.3	5.3 (2.7	6.2 (2.4)	0.039 *	0.0–1.8
QoR_POD1_ mean (± SD)	100 (25)	93 (23)	0.018 *	1–13	102 (24)	94 (23)	0.048 *	0–17
PPOP_POD90rest_ NRS (± SD)	0.6 (1.4)	0.9 (1.7)	0.146	−0.1–0.7	0.7 (1.5)	0.8 (1.7)	0.593	−0.4–0.7
PPOP_POD90movement_ NRS (± SD)	0.7 (1.7)	1.0 (1.9)	0.267	−0.2–0.7	0.8 (1.8)	0.9 (2.0)	0.831	−0.6–0.7
PPOP_POD180rest_ NRS (± SD)	0.3 (1.1)	0.5 (1.4)	0.264	−0.1–0.5	0.4 (1.1)	0.3 (1.0)	0.990	−0.4–0.4
PPOP_POD180movement_ NRS (± SD)	0.5 (1.3)	0.6 (1.5)	0.335	−0.2–0.5	0.4 (1.1)	0.5 (1.3)	0.562	−0.3–0.5
Postoperative opioids MME Mg (± SD)	10.0 (8.3)	17.5 (5.9)	<0.001 **	5.8–9.2	15.2 (6.5)	17.5 (6.5)	0.048 *	0.1–4.5

Abbreviations: APOP; Acute postoperative pain, MME; Morphine milligram equivalents, NRS; Numeric rating scale, POD; Postoperative day, PPOP; Persistent postoperative pain, QoR; Quality of recovery, SD; Standard deviation. Statistically significant difference is marked by an asterisk (*) in conjunction with the absolute value. * Significance at 0.05 level. ** Significance at 0.01 level.

**Table 3:** Stepwise multivariate logistic regression for moderate to severe APOP in the PACU as dependent variable, with predefined variables in terms of clinical significance and earlier scientific merits (PCS).

Predictor	Univariate OR (95% CI)	*p*-Value	Multivariate OR (95% CI)	*p*-Value
VCP ≥ 2.0	1.76 (1.02–3.04)	0.043 *	1.31 (0.73–2.36)	0.361
Current pain	1.22 (1.03–1.45)	0.022 *	1.06 (0.88–1.27)	0.551
Female	3.21 (1.75–5.89)	<0.001 *	2.65 (1.33–5.29)	0.006 **
Age	0.98 (0.97–0.99)	0.039 *	1.00 (0.98–1.02)	0.699
PCS	1.04 (1.01–1.07)	0.019 *	1.03 (0.99–1.06)	0.133
BMI	1.01 (0.98–1.03)	0.675	-	-
Preoperative pain	2.29 (1.32–3.98)	0.003 *	1.76 (0.95–3.24)	0.071

Significance level for univariate logistic regression *p* < 0.1 are highlighted in bold with single asterisk (*). For the multivariate model, only female gender acted as an independent risk factor for moderate to severe APOP (NRS ≥ 4) in the PACU. Significance level of *p* < 0.05 are highlighted as bold and with double asterisk (**).

**Table 4:** Stepwise multivariate logistic regression for VCP ≥ 2.0 as a dependent variable, with predefined variables in terms of clinical significance and earlier scientific merits (PCS).

Variable	Univariate OR (95% CI)	*p*-Value	Multivariate OR (95% CI)	*p*-Value
Current pain	1.28 (1.14–1.44)	<0.001 *	1.23 (1.05–1.43)	0.008 **
Female gender	2.65 (1.56–4.52)	0.003 *	2.25 (1.11–4.55)	0.024 **
Age	0.99 (0.97–0.99)	0.068 *	0.99 (0.98–1.02)	0.868
PCS	1.03 (1.00–1.05)	0.050 *	1.02 (0.99–1.04)	0.279
BMI	0.99 (0.97–1.01)	0.501	-	-
Preoperative pain	1.53 (1.02–2.29)	0.084 *	0.98 (0.58–1.72)	0.990

Significance level cut-off for univariate logistic regression *p* < 0.1 are highlighted in bold with a single asterisk (*). For the multivariate model, female gender, and preoperative current pain on the day of surgery influenced VCP ≥ 2.0. Significance level of *p* < 0.05, highlighted as bold and with a double asterisk (**).

## Data Availability

The data presented in this study are available on request from the corresponding author. The data are not publicly available due to institution policy.

## Abbreviations

The following abbreviations are used in this manuscript:

APOP	Acute postoperative pain
MAO	Multimodal anesthesia with opioids
NRS	Numeric rating scale
OFA	Opioid free anesthesia
PCS	Pain catastrophizing scale
POPIT	Perioperative individualization trial
PPOP	Persistent postoperative pain
PACU	Post-anesthesia care unit
POD	Postoperative day
QST	Quantitative sensory testing
SOC	Standard of care
VCP	Venous cannulation pain
VAS	Visual analog scale
